# Publisher Correction: Artificial light-driven ion pump for photoelectric energy conversion

**DOI:** 10.1038/s41467-019-08815-9

**Published:** 2019-02-14

**Authors:** Kai Xiao, Lu Chen, Ruotian Chen, Tobias Heil, Saul Daniel Cruz Lemus, Fengtao Fan, Liping Wen, Lei Jiang, Markus Antonietti

**Affiliations:** 1 Department of Colloid Chemistry, Max Planck Institute of Colloids and Interfaces, 14476 Potsdam, Germany; 2 Key Laboratory of Bio-inspired Smart Interfacial Science and Technology of Ministry of Education, School of Chemistry, Beihang University, Beijing, 100191 PR China; 3 State Key Laboratory of Catalysis, 2011-iChEM, Dalian National Laboratory for Clean Energy (DNL), Dalian Institute of Chemical Physic (DICP), Zhongshan Road 457, Dalian, 116023 PR China; 4 Key Laboratory of Bio-inspired Materials and Interfacial Science, Technical Institute of Physics and Chemistry, Chinese Academy of Sciences, Beijing, 100190 PR China; 5 School of Future Technologies, University of Chinese Academy of Sciences, Beijing, 101407 PR China

Correction to: *Nature Communications*; 10.1038/s41467-018-08029-5; published online 08 January 2019.

The original version of this Article contained errors in Fig. 3. In Fig. 3d, the label ‘With light irradiation’ was originally incorrectly given as ‘Without light irradiation’. In Fig. 3f, the label ‘Without light irradiation’ was originally incorrectly given as ‘With light irradiation’. This has been corrected in both the PDF and HTML versions of the Article.

