# Supplementary material for: Artificial light-driven ion pump for photoelectric energy conversion
Source: Nat Commun. 2019 Jan 8;10:74. doi: 10.1038/s41467-018-08029-5 (PMC6325115; doi:10.1038/s41467-018-08029-5)
Supplement: Supplementary file 1 — Supplementary Information [file 41467_2018_8029_MOESM1_ESM.pdf]

Supplementary information for

Artificial light-driven ions pump for photoelectric energy conversion

Kai Xiao et al.

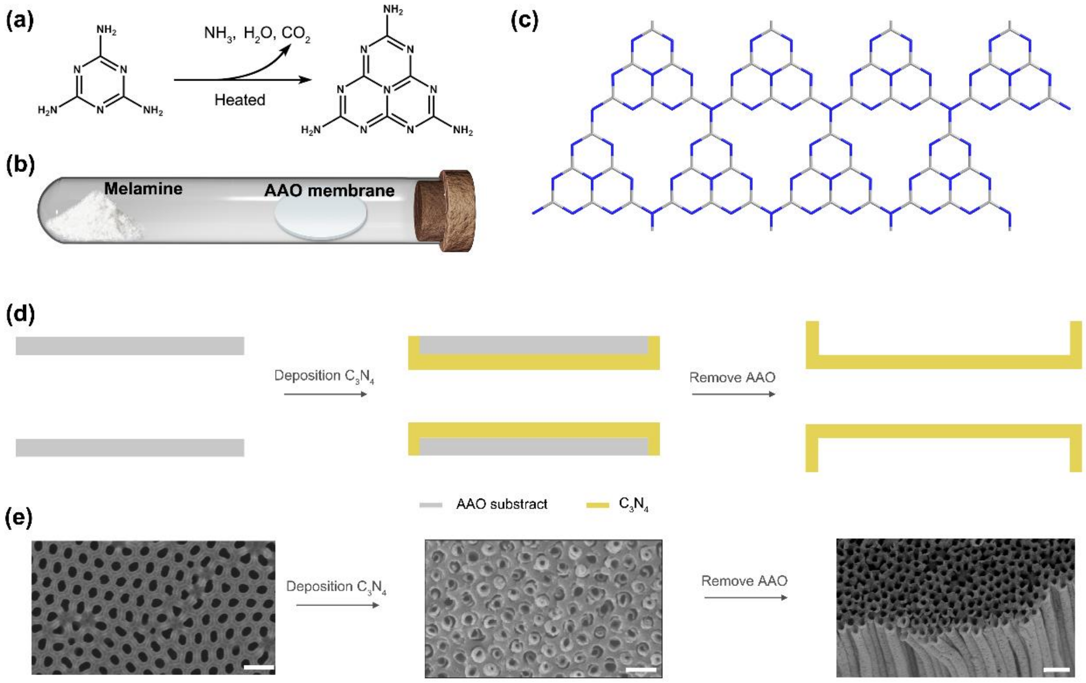

Supplementary Figure 1 | CNNM fabrication. a, The synthetic route of CNNM. **b,** Schematic illustration of the vapour-deposition nanotube. **c**, The molecule structure of carbon nitride. **d**, Schematic illustration of the vapour-deposition process and removing AAO substrate. **e**, SEM images of bare AAO substrate, carbon nitride nanotube inserting AAO and carbon nitride nanotube after removing AAO, scale bar 200 nm.

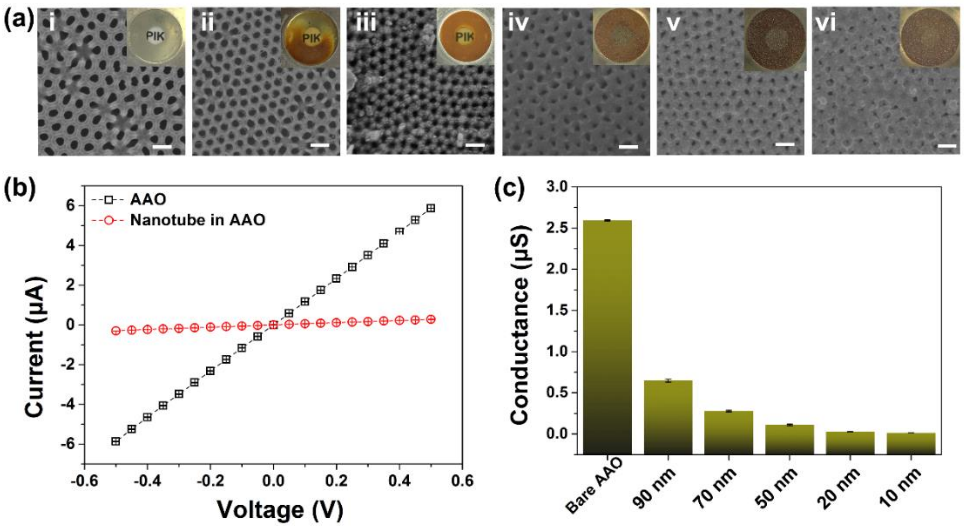

Supplementary Figure 2 | CNNM geometry. a, The CNNM with different tube diameters from about 100 nm to 10 nm, scale bar 200 nm. **b,** The current-voltage curves of AAO and CNNM with 20 nm inner pore diameter. **c**, The conductance of CNNMs as a function of nanotube diameter. Error bars represent standard deviations of independent triple experiments.

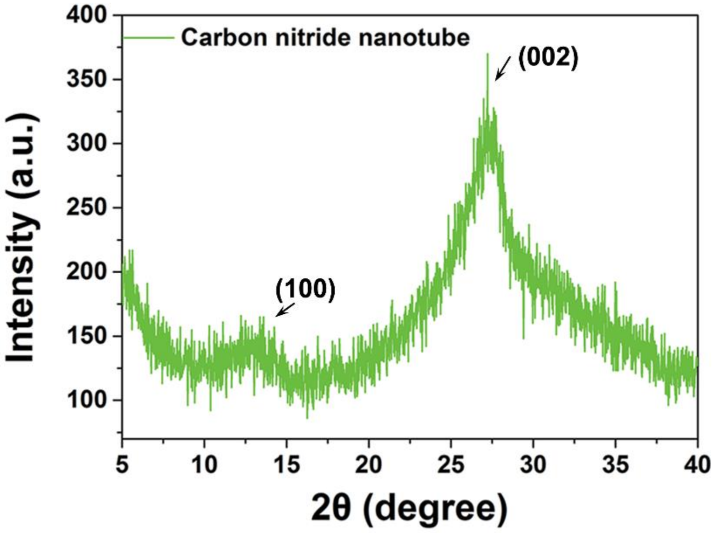

Supplementary Figure 3 | XRD of carbon nitride nanotube. The 100 and 002 peaks are consistent with previous work.[1,2]

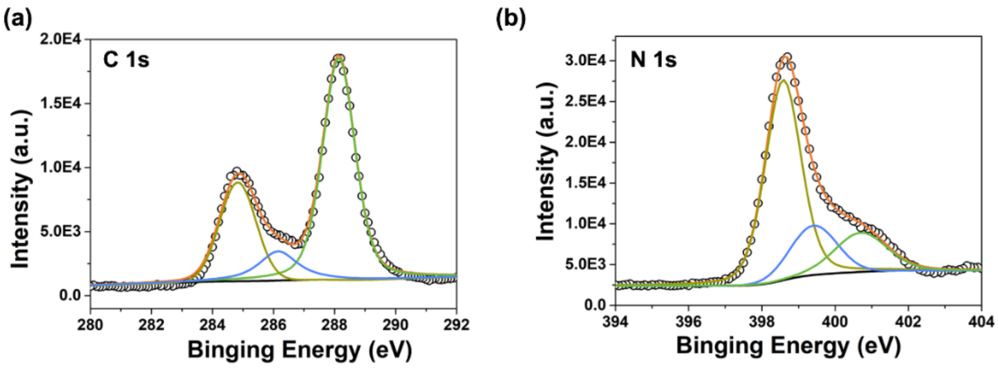

Supplementary Figure 4 | XPS of carbon nitride nanotube. High-resolution C 1s (a) spectra of carbon nitride nanotube, indicating two typical C 1s peaks at 284.8 and 288.1 eV that can be assigned to the sp^2 C in carbonaceous environment and sp^2 C in C-N heterocycles, respectively. The N 1s spectra (b) can be deconvoluted into three peaks, 398.6 eV (C=N-C), 399.9 eV ($N-C_3$), and 400.9 eV (C-NH-C and $C-NH_2$), consistent with previous work.[3]

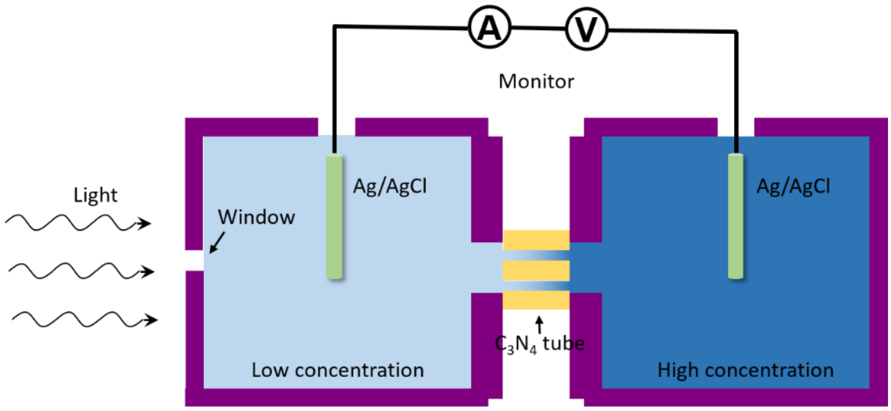

Supplementary Figure 5 | Schematic of the test device.

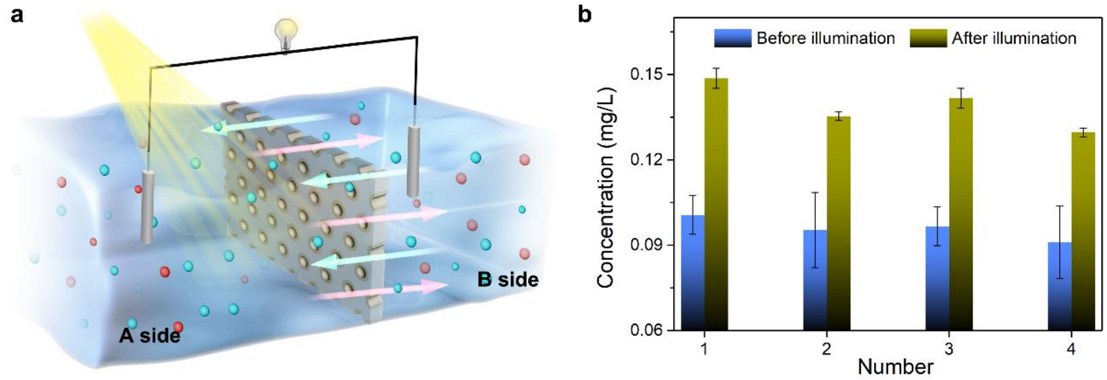

Supplementary Figure 6| Concentration of K^+ ions before and after "pump process". (a) Schematic of photo-induced ions pump process, the initial concentration of A and B side are the same (about 0.000002 M/L). (b) Concentration of K^+ ions measured by inductively coupled plasma (ICP) before and after pump process (four parallel results). The result clearly indicated that the concentration increased after 200 s light illumination. Error bars represent standard deviations of independent triple experiments.

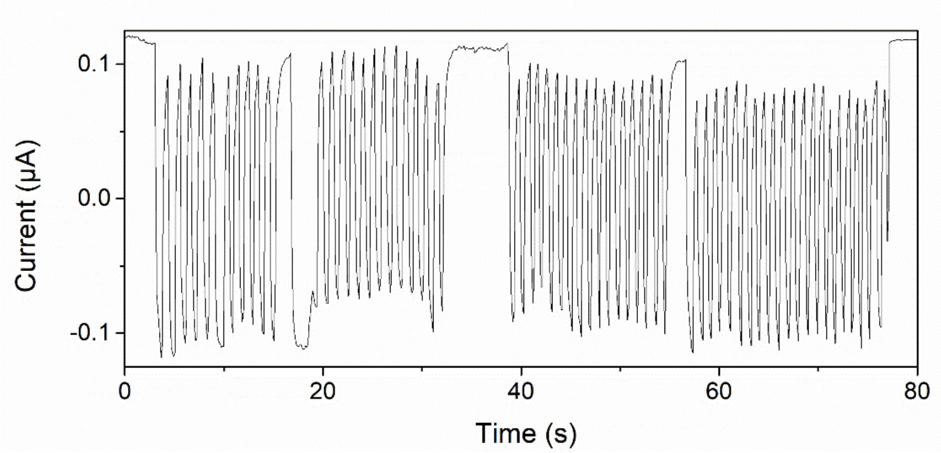

Supplementary Figure 7 | Ions pump properties with various interval frequency. This result shows that the CNNM-based ions pump response to light very fast.

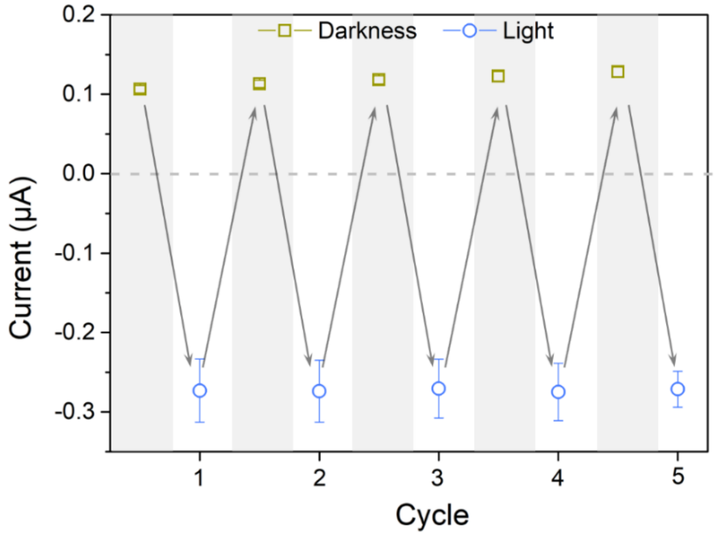

Supplementary Figure 8 | Reversibility of ions pump. This result shows ions pump is reversible and stable under several cycles of illumination. The interval for each cycle is 30 min.

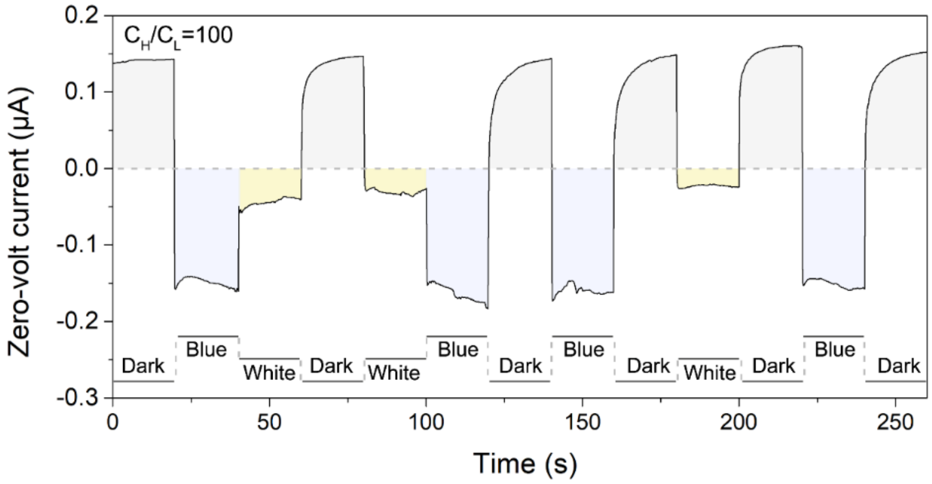

Supplementary Figure 9 | Spectral dependence of ion pump. The CNNM-based ion pump can work in blue light (465±1.0 nm) and solar light, while blue light can provide more "pump energy" than solar light with the same light intensity because the strongest absorption of CNNM is in 420 nm. In this work, we mainly use white light to mimic sunlight. Therefore, we can get higher performance ion pump and energy conversion properties by using blue light.

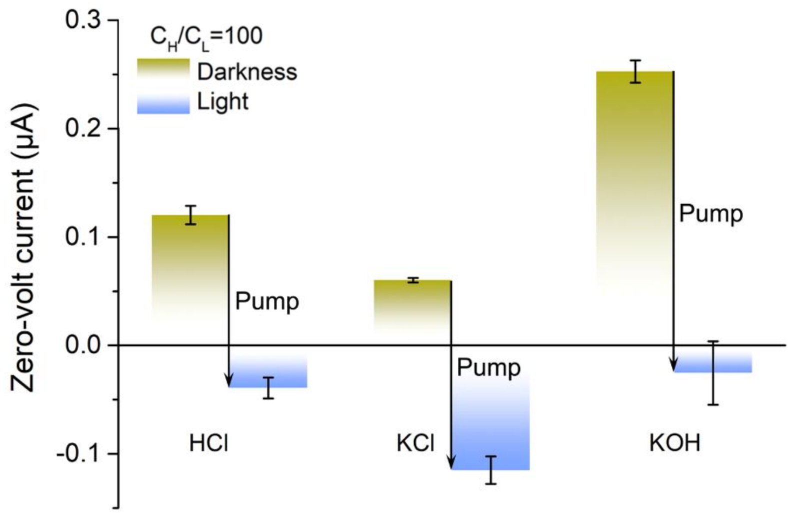

Supplementary Figure 10 | pH dependence of ion pump. The CNNM ion pump is effective in different electrolyte solutions: acid solution (HCl), saline solution (KCl), and alkali solution (KOH). Error bars represent standard deviations of independent triple experiments.

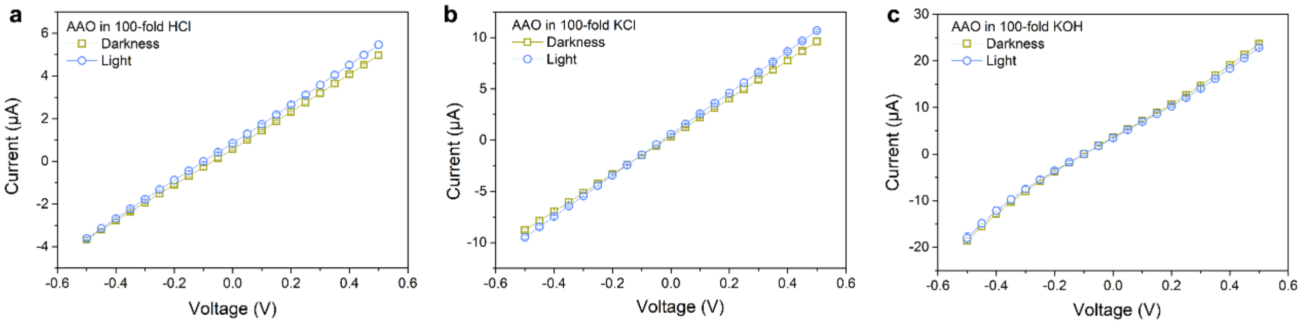

Supplementary Figure 11 | Current-voltage curves of AAO membrane in different electrolyte solutions before and after illumination. **a**, acid solution (HCl). **b**, saline solution (KCl) **c**, alkali solution (KOH). There is no obvious difference before and after light illumination for naked AAO membrane.

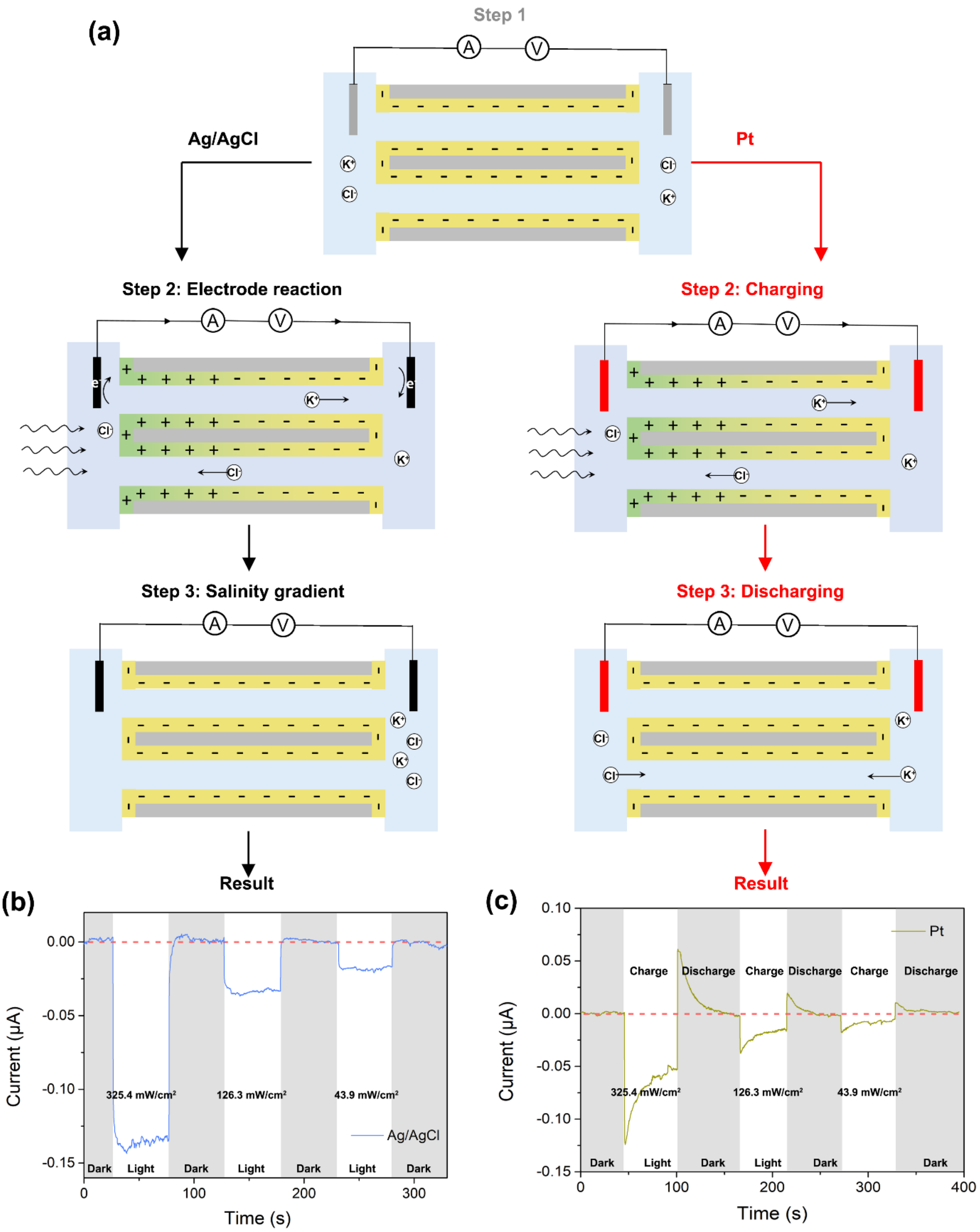

Supplementary Figure 12 | Ions transport with different electrodes. a. Schematic of ions transport before and after light illumination with Ag/AgCl (left part) and Pt (right part) electrodes respectively. **b.** Photo-induced ionic current as a function of the light intensity with Ag/AgCl as electrode. **c.** Photo-induced charging and discharging process as a function of the light intensity with Pt as electrode. These results indicate that surface charge redistribution of CNNM due to the photo-induced separation of electrons and holes is thought to be key to the ion pump phenomenon.

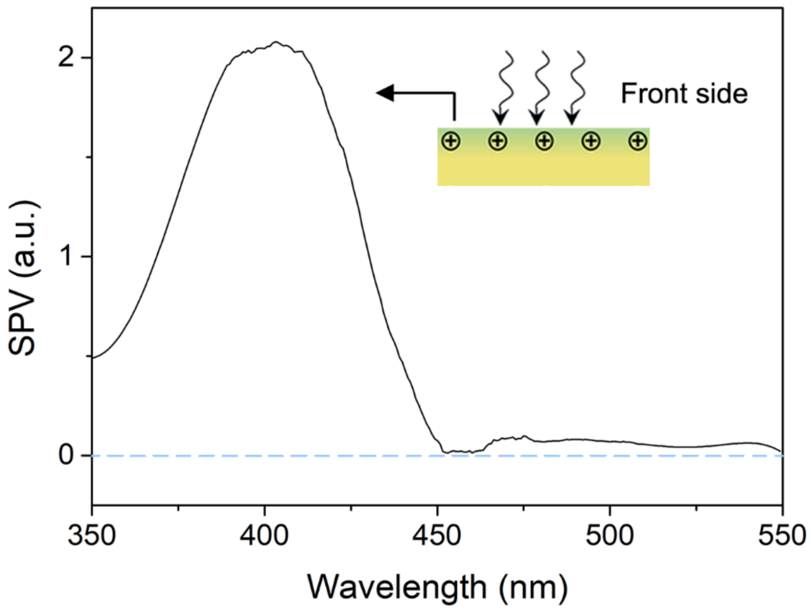

Supplementary Figure 13 | Surface photo voltage spectrum. Surface photo voltage spectrum (SPV) of the CNNM shows the illuminated side (front side) is positive charged.

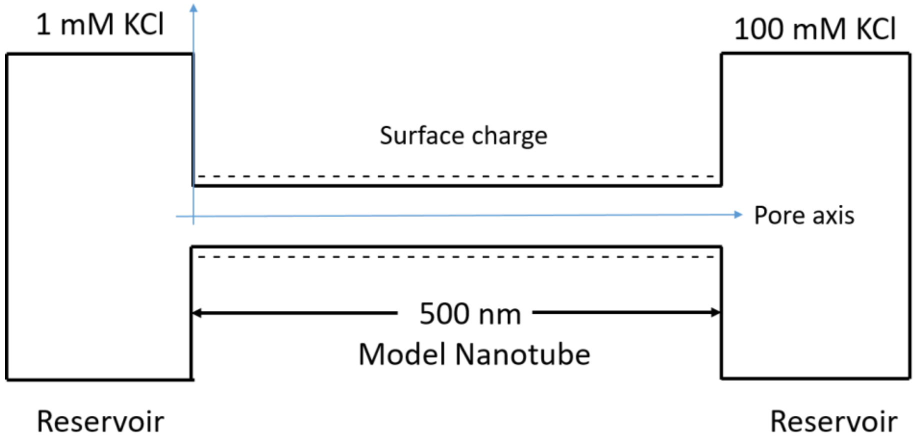

Supplementary Figure 14 | Calculation nanotube model (not in scale). The theoretical simulation is based on the coupled two-dimensional Poisson–Nernst–Planck equations within the commercial finite-element package COMSOL 5.1 script environment.

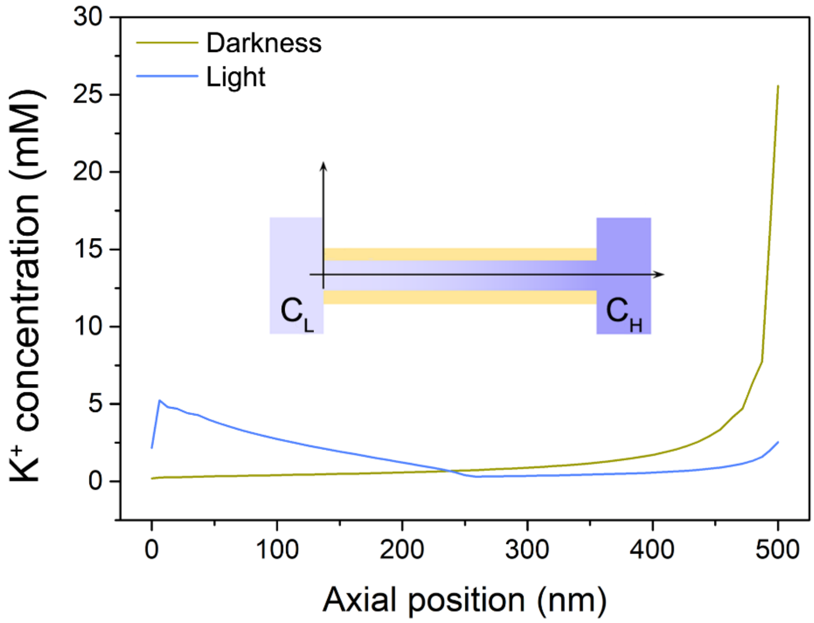

Supplementary Figure 15 | Calculated K^+ ions distribution in the nanotube before and after illumination. Before illumination, the K^+ ions concentration in C_H side is much higher than C_L side, while the Cl^- ions concentration in C_L side is a little higher than C_H side after illumination, indicating the ions pump phenomenon in CNNM.

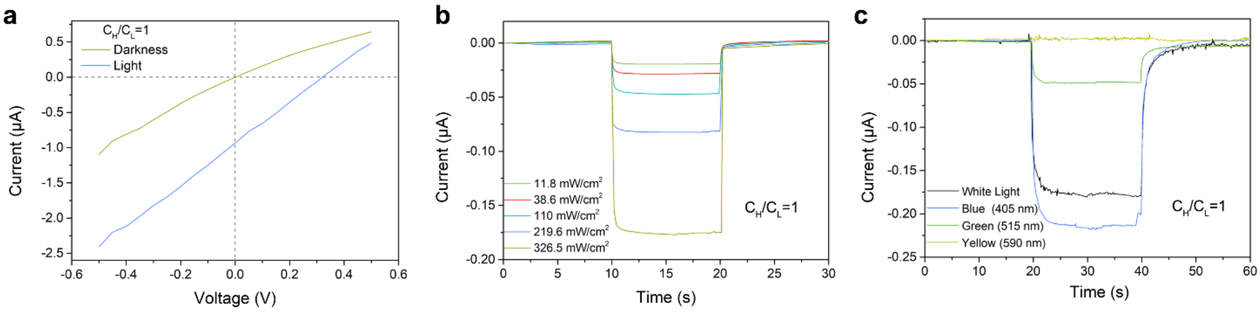

Supplementary Figure 16 | Light-induced ion pump phenomenon under symmetric electrolytes concentration. a. The typical current-voltage curves before and after light (143 mW/cm^2) irradiation without concentration gradient (C_H=C_L=0.01 M KCl). **b.** The current as a function of light density from 11.8 mW/cm^2 to 326.5 mW/cm^2 without concentration gradient (C_H=C_L=0.01 M KCl) under 0 V bias. **c.** The current as a function of light wavelength without concentration gradient (C_H=C_L=0.01 M KCl) under 0 V bias.

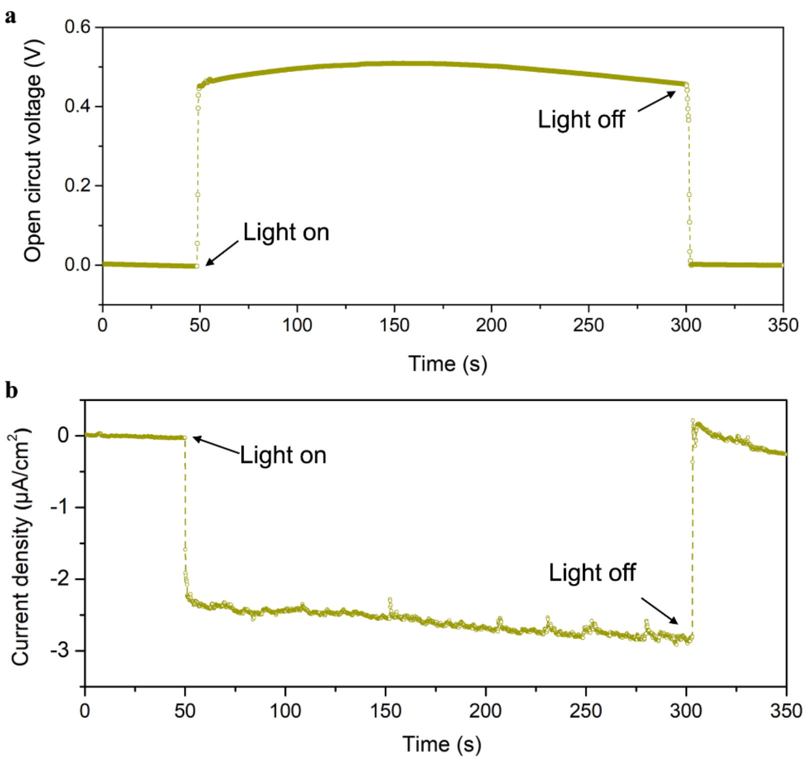

Supplementary Figure 17 | Stability of photoelectric energy conversion system. a,**b**, Both the photocurrent (a) and photovoltage (b) did not show any sign of deterioration under long continuous illumination.

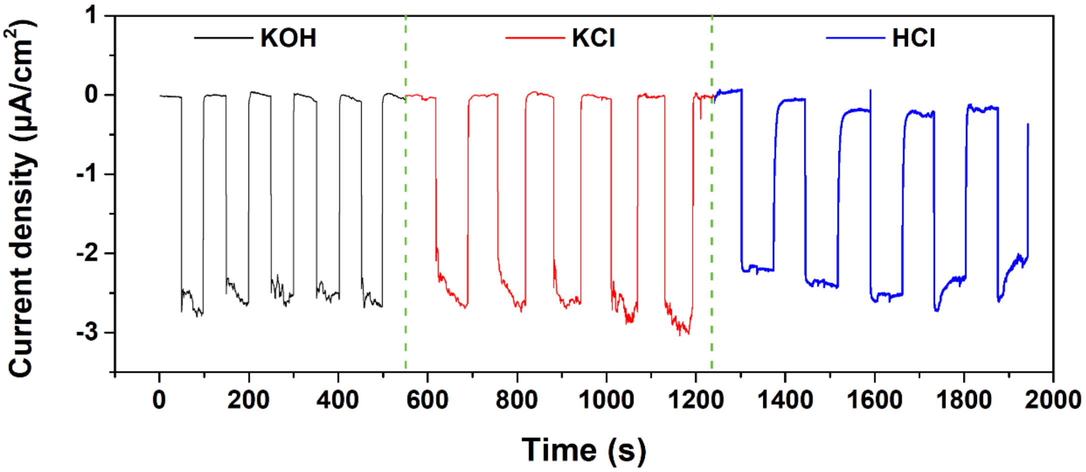

Supplementary Figure 18 | pH dependence of photoelectric energy conversion system. The CNNM ion pump is effective to harvest light energy in different electrolyte solutions: acid solution (HCl), saline solution (KCl), and alkali solution (KOH).

Supplementary methods

Experimental section:

Unless otherwise noted, all of the commercial reagents were used as received. Melamine (purity >98.0%) was purchased from Sigma-Aldrich. Target 60-μm-thick AAO membrane with pore width 84 ± 16 nm were purchased from Heifei Puyuan Nano, China. Glass test tube for vaper-deposition polymerization (VDP) were purchased from Merck Millipore. LED lights (White, Blue, Green, Yellow) were used for photo-induced ions transport evaluation, respectively. In this work, unless otherwise noted, all the light illumination were provided by white LED light, which can mimic sunlight. The *I-V* curves were adjusted to zero current at zero voltage to remove small offsets experienced between runs. All measurements were carried out at room temperature. The main transmembrane potential used in this work was stepped at 0.1 V/step for 1 s/step (0.1 V/s) from -1 to +1 V, with its period of 21 s.

Characterizations

The released carbon nitride nanotubes were transferred to a quartz glass substrate and analyzed. X-ray photoelectron spectroscopy (XPS) was performed by an ESCALab220i-XL electron spectrometer from VG Scientific using 300W Al Kα radiation, and the base pressure was about 3×10^{-9} mbar. The binding energies were referenced to the C1s line at 284.8 eV from adventitious carbon. The scanning electron microscope (SEM) JSM-7500F (JEOL) at an accelerating voltage of 3 kV was used to get the top view and cross section of the CNNM. The TEM is a double-corrected Jeol ARM200F, equipped with a cold field emission gun. The acceleration voltage was set to 200kV and the emission was put to 10μA. The K^+ ions concentrations before and after illumination are measured by ICP-Optical Emission Spectrometer (Optima 800, PerkinElmer). X-ray diffraction (XRD) patterns were recorded with a Bruker D8 Advance instrument with Cu Kα radiation. Shimadzu UV 2600 was used to reveal the optical absorbance spectra of CNNM and powders. Surface photovoltaic spectroscopy was measured by a surface photovoltaic spectrometer (CEL-TPV1000). The Zeta Potential was measured with SurPASS 3, Anton Paar.

PNP calculation.

The trans-nanotube potential was systematically analyzed by a theoretical model based on Poisson and Nernst-Planck (PNP) equations with proper boundary conditions,[4,5]

$$\Delta^2\Phi = -\frac{F}{\varepsilon}\sum z_i c_i$$

$$j_i = D_i \Delta c_i + \frac{z_i F}{RT} D_i c_i \Delta\Phi$$

$$\Delta \cdot j_i = 0$$

whereΦ, c_i, D_i, j_i, z_i are, respectively, the electrical potential, ion concentration, diffusion constant, ionic flux, and charge of species i. ε is the dielectric constant of the electrolyte solution. The diffusion coefficients for cations and anions are 2.0×10^{-9} m^2/s (we use KCl electrolyte for simplicity). The boundary condition for potential Φ on the nanotube wall is,

$$\vec{n}\cdot\nabla\Phi = -\frac{\sigma}{\varepsilon}$$

where σ is the surface charge density. And the surface charge density is various along with our experiment condition. The ion flux has the zero normal components at boundaries,

$$\vec{n}\cdot j = 0$$

The geometric parameters are in supporting figure 14. To carry out the calculations, the Comsol Multiphysics 5.1 was used with the “electrostatics (Poisson equation)” and “Nernst-Planck without electroneutrality” modules. The stationary solver was generally used. But when it fails, the parametric solver was applied. For all the calculations, the accuracy is set to be less than 10^{-6}.

Supplementary references

1 Poisson-Nernst-Planck model of ion current rectification through a nanofluidic diode. *Phys. Rev. E* **76**, 041202 (2007).

2 Ion current rectification at nanopores in glass membranes. *Langmuir* **24**, 2212-2218 (2008).

3 Condensed Graphitic Carbon Nitride Nanorods by Nanoconfinement: Promotion of Crystallinity on Photocatalytic Conversion. *Chem. Mater.* **23**, 4344-4348 (2011).

4 Graphitic Carbon Nitride (g-C3N4)-Based Photocatalysts for Artificial Photosynthesis and Environmental Remediation: Are We a Step Closer To Achieving Sustainability? *Chem. Rev.* **116**, 7159-7329 (2016).

5 Compact carbon nitride based copolymer films with controllable thickness for photoelectrochemical water splitting. *J. Mater. Chem. A* **5**, 19062-19071 (2017).
